# Characterization of the Impact of Daclizumab Beta on Circulating Natural Killer Cells by Mass Cytometry

**DOI:** 10.3389/fimmu.2020.00714

**Published:** 2020-04-24

**Authors:** Thanmayi Ranganath, Laura J. Simpson, Anne-Maud Ferreira, Christof Seiler, Elena Vendrame, Nancy Zhao, Jason D. Fontenot, Susan Holmes, Catherine A. Blish

**Affiliations:** ^1^Department of Medicine, Stanford University School of Medicine, Stanford, CA, United States; ^2^Department of Statistics, Stanford University, Stanford, CA, United States; ^3^Biogen, Cambridge, MA, United States; ^4^Chan Zuckerberg Biohub, San Francisco, CA, United States

**Keywords:** natural killer cell, daclizumab beta, multiple sclerosis, immune profiling, CyTOF/mass cytometry, uniform manifold approximation and projection, CytoGLMM, clustering

## Abstract

Daclizumab beta is a humanized monoclonal antibody that binds to CD25 and selectively inhibits high-affinity IL-2 receptor signaling. As a former treatment for relapsing forms of multiple sclerosis (RMS), daclizumab beta induces robust expansion of the CD56^bright^ subpopulation of NK cells that is correlated with the drug’s therapeutic effects. As NK cells represent a heterogeneous population of lymphocytes with a range of phenotypes and functions, the goal of this study was to better understand how daclizumab beta altered the NK cell repertoire to provide further insight into the possible mechanism(s) of action in RMS. We used mass cytometry to evaluate expression patterns of NK cell markers and provide a comprehensive assessment of the NK cell repertoire in individuals with RMS treated with daclizumab beta or placebo over the course of 1 year. Treatment with daclizumab beta significantly altered the NK cell repertoire compared to placebo treatment. As previously reported, daclizumab beta significantly increased expression of CD56 on total NK cells. Within the CD56^bright^ NK cells, treatment was associated with multiple phenotypic changes, including increased expression of NKG2A and NKp44, and diminished expression of CD244, CD57, and NKp46. These alterations occurred broadly across the CD56^bright^ population, and were not associated with a specific subset of CD56^bright^ NK cells. While the changes were less dramatic, CD56^dim^ NK cells responded distinctly to daclizumab beta treatment, with higher expression of CD2 and NKG2A, and lower expression of FAS-L, HLA-DR, NTB-A, NKp30, and Perforin. Together, these data indicate that the expanded CD56^bright^ NK cells share features of both immature and mature NK cells. These findings show that daclizumab beta treatment is associated with unique changes in NK cells that may enhance their ability to kill autoreactive T cells or to exert immunomodulatory functions.

## Introduction

Natural killer (NK) cells are innate lymphocytes that are best known for the eponymous function: that of killing other cells. Yet NK cells also play a critical role in immune regulation by secreting cytokines that influence the character of the immune response. NK cell function is controlled by signals received through activating, inhibitory and cytokine receptors (1). Activating receptors, examples of which include the natural cytotoxicity receptors NKp30, NKp44, and NKp46, the C-type lectin receptors NKG2D and NKG2C, and certain classes of activating Killer-cell Immunoglobulin-like Receptors (KIRs), generally sense stress on the target cell and promote NK cell activation. Inhibitory receptors, including most KIRs, NKG2A, and LILRB1 (CD85j), generally recognize Major Histocompatibility Class (MHC) I receptors and dampen NK cell responses to normal healthy cells. Broadly, NK cells are divided into two major classes. In the blood, the mature CD56^dim^ NK cells are the predominant subset and have potent cytotoxic activity, while the relatively immature CD56^bright^ NK cells are generally present at <10% and primarily secrete cytokines. Recent studies have demonstrated significant heterogeneity in the human NK cell repertoire, with a wide range of NK cell subsets expressing different combinations of these activating and inhibitory receptors (2–6).

Natural killer cells also express a wide range of cytokine receptors making them extremely responsive to cytokine stimulation. NK cells undergo dramatic shifts in phenotype and function in the presence of cytokines such as IL-2, IL-12, IL-15, and IL-18, singly and in combination (7–9). IL-2 plays a particularly critical role in activating NK cells by binding to the low affinity IL-2 receptor, a heterodimer of CD122 (IL-2Rβ) and CD132 (IL-2Rγ, otherwise known as the common gamma chain). In general, NK cells do not express the high affinity IL-2 receptor, CD25 (IL-2Rγ). The CD56^bright^ NK cell subset expresses much higher levels of CD122 than the CD56^dim^ subset (10–12).

Daclizumab is a humanized monoclonal antibody that irreversibly blocks CD25, preventing signaling through the high affinity IL-2R while increasing IL-2 bioavailability to bind to the low affinity receptor [reviewed in (13, 14)]. Due to the complex roles of IL-2 *in vivo*, daclizumab induces several immunological changes, including inhibition of T cell activation, reduction in the frequency and survival of regulatory T cells, and expansion of CD56^bright^ NK cells (13, 14). It was originally developed as an intravenous treatment for several disease indications, including the prevention of transplant rejection and the treatment of severe uveitis and T cell leukemia (13, 14). Later a subcutaneous form (daclizumab beta) was developed and approved for the treatment of relapsing forms of multiple sclerosis (RMS) due to its beneficial effects including reduction in lesion size and slowed disease progression (10, 15–18); (19). In these initial trials, daclizumab beta treatment was associated with adverse events including cutaneous reactions, malignancies, infections, and transaminase elevations, though these were not sufficiently severe to preclude approval. In 2018, daclizumab beta was voluntarily withdrawn from the market due to the nature and complexity of adverse events associated with the drug and limited number of patients treated, which presented challenges in further characterizing its evolving benefit/risk profile. Subsequently, cases of immune-mediated encephalitis were confirmed as adverse drug reactions that can be related to treatment with daclizumab beta.

While no longer used therapeutically, a better understanding of the effects of daclizumab beta may provide insight into the pleiotropic effects of IL-2 in the setting of RMS. Surprisingly, the beneficial effects of daclizumab beta treatment were linked not to changes in T cell function, but instead were strongly correlated with expansion of CD56^bright^ NK cells (10, 13, 14, 16). Although CD56^bright^ NK cells generally have poor cytotoxic activity, the daclizumab beta-expanded CD56^bright^ NK cells could kill activated, autologous CD4^+^ T cells, potentially driving the therapeutic effect by eliminating autoreactive T cells (10, 20). This study was undertaken to provide a better understanding of the effects of daclizumab beta on circulating NK cells *in vivo*.

## Materials and Methods

### Study Subjects

Cryopreserved peripheral blood mononuclear cells (PBMCs) from daclizumab beta-treated and placebo-treated individuals living with RMS were chosen from the Biogen SELECT (NCT00390221) and DECIDE (NCT01064401) studies (19, 21). Subjects were treated subcutaneously with 150 mg daclizumab beta every 4 weeks for 52 weeks. For the placebo and the treatment cohort, we received de-identified PBMCs at 3 timepoints: Baseline, Week 24 and Week 52. For the healthy donor cohort, leukoreduction system chambers from anonymous donors were purchased from the Stanford Blood Bank. PBMCs were isolated by Ficoll density gradient centrifugation and then cryopreserved in fetal bovine serum (FBS) with 10% dimethyl sulfoxide (DMSO). We had 16 healthy donors, 17 placebo and 30 daclizumab beta treated individuals. As part of their initial enrollment, all subjects provided written informed consent. The studies were approved by the relevant central and local ethics committees and were conducted in accordance with the International Conference on Harmonization guidelines for Good Clinical Practice and the principles of the Declaration of Helsinki.

### Antibody Conjugation, Mass Cytometry Staining and Data Acquisition

Antibodies for mass cytometry were conjugated to heavy metals using MaxPar^®^ × 8 labeling kits (Fluidigm) as described (22). To ensure antibody stability over time, the antibody panel was lyophilized into single-use pellets prior to use (Biolyph). PBMCs were thawed at 37°C in RPMI-1640 media (supplemented with 10% FBS, L-glutamine, and Penicillin-Streoptomycin-Amphotericin) with benzonase. NK cells were purified by magnetic bead isolation via negative selection (Miltenyi, cat. 130-092-657) and stained with the NK cell antibody panel (Supplementary Table S1) as previously described (4, 23, 24). Cells were resuspended in 1x EQ Beads (Fluidigm) for normalization before acquisition on a Helios mass cytometer (Fluidigm).

### Data Analysis

The open source statistical software R^1^ was used for all statistical analyses (25). Signal intensities were transformed using the hyperbolic sine transformation (asinh function) prior to statistical analysis, with cofactor equal to 5, to account for heteroskedasticity. We used the custom-made package *CytoGLMM* (26, 27) to identify markers predictive of a given sample type while taking into account the subject effect. To this end, this package uses a generalized linear mixed model with paired comparison (used for analyses of the same individual over time) and generalized linear model with bootstrap resampling (for cross-sectional comparisons between daclizumab beta- and placebo-treated individuals). Using the empirical marker distribution, the model generates the log-odds that the expression of a given marker is predictive of the sample type (for example, drug-treated vs. placebo-treated) with the 95% confidence intervals. For paired comparisons, we computed *p*-values using the asymptotic theory implemented in R package *mbest* (28). For unpaired comparisons, we computed *p*-values by inverting the percentile bootstrap confidence intervals and assuming two-sided intervals with equal tails (29). To correct for multiple comparisons, the Benjamini-Hochberg method controlling the False Discovery Rate (FDR) at level 0.05 was used, which is conservative as it assumes independence of markers. For paired analysis on daclizumab beta-treated individuals over time, where the response variable was timepoint, we used all cells from each donor. For unpaired analysis using the bootstrap, where the response variable was daclizumab beta or placebo treatment, we used 1,000 cells from each sample for the total NK cell and CD56^dim^ analyses, and used all cells from each sample for CD56^bright^. There were fewer than 1,000 CD56^bright^ NK cells in most samples except for daclizumab beta treated individuals. The number of subjects used for each analysis is specified in the figure legends.

### UMAP Visualizations

The Uniform Manifold Approximation and Projection (UMAP) algorithm was used as a visualization and dimensionality reduction technique for our CyTOF data (30, 31). The *uwot* R package provides an implementation of UMAP and was used with a minimum distance set to 0.1 and nearest neighbors set to 20. The UMAP loadings were visualized using Cytobank. Separate analyses were performed on total NK cells and CD56^bright^ NK cells, including both placebo and drug treatment at three different timepoints. All markers in Supplementary Table S1 were used excluding markers used for gating (CD3, CD19, CD33, CD14, CD56, CD4), and markers with extremely low or non-specific staining (FcRγ, Ki-67, KIR2DS2, CXCR6, PD1).

### Clustering and Differential Abundance Tests

We used a clustering method to identify subsets of cells in the NK and CD56^bright^ cell populations in the placebo and daclizumab beta treated individuals. The clustering analysis was performed using the CATALYST package version 1.10.0 [Crowell et al. (32) CATALYST: Cytometry dATa anALYSis Tools] from Bioconductor. The clustering method provided by the package combines two algorithms. The first step uses the FlowSOM clustering algorithm (33) to cluster the data into 100 high-resolution clusters. The second step regroups these clusters into metaclusters using the ConsensusClusterPlus metaclustering algorithm (34). The default parameters of the cluster function were used except for the maximum of metaclusters which was defined to 30. The delta area plot provided by the package was used to select the optimal number of metaclusters (9 for the CD56^bright^ cell population; 5 for the NK cell population).

We performed differential abundance tests to highlight differences in cell clusters due to the Daclizumab beta treatment. The differential abundance tests were performed with the diffcyt package version 1.6.0 (35). The diffcyt-DA-edgeR method uses the edgeR package (36) which fits a negative bionomial generalized linear model to identify populations that are present at different frequencies. For each test, we filtered the data to the comparison of interest. We created the design matrix corresponding to the experimental design and contrast matrix specifying the comparison of interest. The differential abundance test reports adjusted *p*-values (FDR).

### Data Availability

The dataset generated and analyzed for this study can be found in FlowRepository ID FR-FCM Z2D6.

## Results

### Characteristics of Study Population

For this study, individuals living with RMS received 150 mg daclizumab beta or placebo subcutaneously every 4 weeks for 52 weeks. The demographics of the healthy controls, placebo-treated, and daclizumab beta-treated groups are given in Table 1. As expected with RMS, we had a high frequency of females in the trial, with 90% in the daclizumab beta-treated group and 70% in the placebo group. Peripheral blood samples for our research study were taken from both the SELECT and DECIDE trials (19, 21).

**TABLE 1:** Demographics of the study population.

Group	Total *N*	Pct. Female	Age, years: median (range)
Healthy	16	50%	52.5 (27–82)
Placebo	17	70.5%	34 (21–45)
Daclizumab beta	30	90%	32 (19–52)

### Characterization of the NK Repertoire

Frozen peripheral blood samples from baseline (pre-treatment), 24 weeks post-treatment initiation, and 52 weeks post-treatment initiation were obtained for this study. Purified NK cells from each sample were stained for mass cytometry using a panel of 41 antibodies conjugated to heavy metals (Supplementary Table S1 and Supplementary Figure S1A). Total NK cells, CD56^bright^ and CD56^dim^ NK cells were analyzed by gating in FlowJo (Supplementary Figure S1B). Example staining of each of the 31 NK markers used in this analysis are shown in Supplementary Figure S2.

### Daclizumab Beta Induces Higher Frequency of CD56^bright^ NK Cells

**FIGURE 1:**
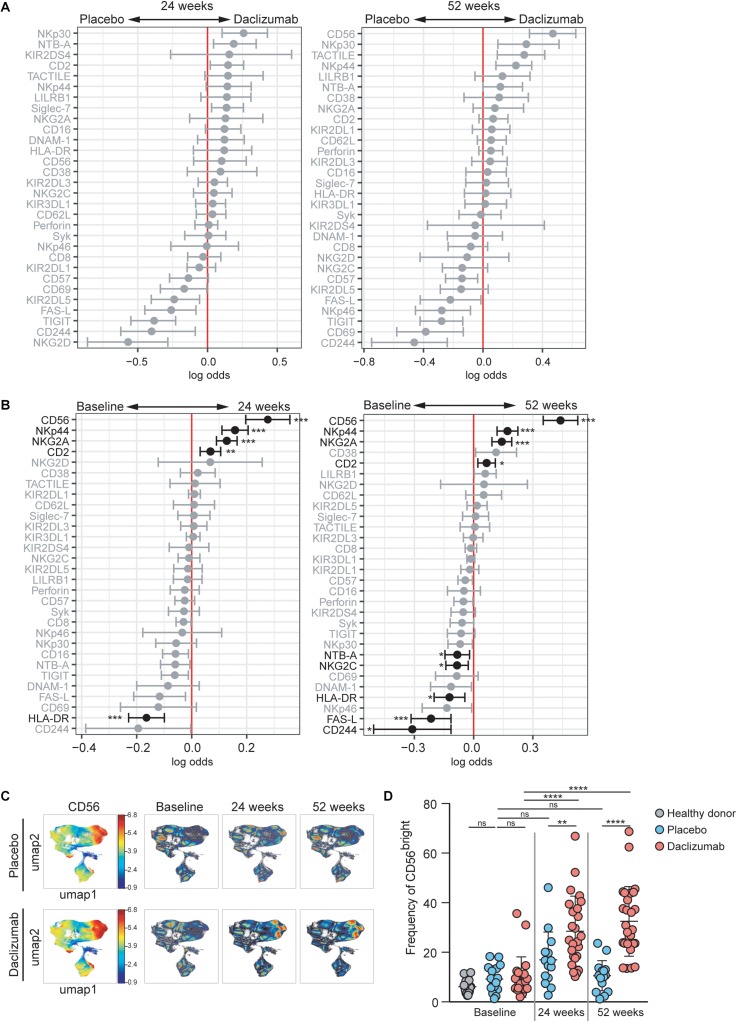
Daclizumab beta induces CD56^bright^ NK cells with a distinct phenotype**. (A)** A generalized linear model with bootstrap resampling was used to identify NK markers predictive of daclizumab beta- and placebo-treated individuals at 24 (left) and 52 (right) weeks of treatment. Log-odds are logarithm of ratios of the probability that a cell belongs to each treatment group. An increase in the parameter coefficient corresponds to the strength of the classification power, with the 95% confidence interval represented by the line surrounding the point estimate. Gray lines indicate markers with adjusted *p*-values > 0.05. Black lines indicate markers with adjusted *p*-values < 0.05. Total NK cells were used, with subsampling to 1000 cells per individual. Placebo: 24 weeks, *n* = 14; 52 weeks, *n* = 16. Daclizumab beta: 24 weeks, *n* = 25; 52 weeks, *n* = 27. **(B)** A generalized linear mixed model with paired comparison was used for analyses of the same individual over time, comparing baseline and 24 weeks of daclizumab beta treatment (left), and baseline and 52 weeks of daclizumab beta treatment (right). Total NK cells were used with no subsampling. Daclizumab beta baseline vs. 24 weeks, *n* = 21. Daclizumab beta baseline vs. 52 weeks, *n* = 22. **(C)** UMAP visualization of all NK cells from placebo and daclizumab beta treatment groups. Panels on the left are colored by CD56 expression. Baseline, 24 weeks, and 52 weeks samples are shown colored by density. **(D)** The frequency of CD56^bright^ NK cells for each individual are shown as a percentage of total NK cells. Healthy donors (*n* = 16, gray), placebo treatment (baseline, *n* = 16; 24 weeks, *n* = 14; 52 weeks, *n* = 16; blue), daclizumab beta treatment (baseline, *n* = 22; 24 weeks, *n* = 25, 52 weeks, *n* = 27; pink). *Adjusted *p*-value < 0.05, **adjusted *p*-value < 0.01, ***adjusted *p*-value < 0.001, ****adjusted *p*-value < 0.0001. Adjusted *p*-values calculated on generalized linear mixed model in **(B)** using Benjamini-Hochberg method with FDR = 0.05. Adjusted *p*-values in **(D)** calculated using one-way ANOVA with Sidak’s multiple comparisons test.

The *CytoGLMM* R package was used to identify which NK markers predicted daclizumab beta treatment compared to placebo. This generalized linear model with bootstrap resampling allows for identification of markers that predict a given outcome, while controlling for inter-individual variability. The model takes into account the full distribution of the marker measurements (rather than a single summary measure such as mean signal intensity) and yields the log-odds with which that marker predicts the outcome, with 95% confidence intervals. Among total NK cells at 24 weeks, NKp30, NTB-A, and CD2 expression predicted daclizumab beta treatment, while NKG2D, CD244, TIGIT, FAS-L, and KIR2DL5 predicted placebo treatment (Figure 1A). After controlling for multiple comparisons, these changes were not statistically significant. At 52 weeks, among the total NK cell population, CD56, NKp30, TACTILE, NKp44, and NTB-A predicted daclizumab beta treatment, while CD244, CD69, TIGIT, NKp46, and CD57 predicted placebo treatment (Figure 1A), but these changes were not significant after correction for multiple comparisons. There were no markers that significantly predicted placebo or daclizumab treatment at baseline, indicating that the groups were relatively well matched, and changes we observe at later time points were not due to baseline differences between the two groups (Supplementary Figure S3A). There were only two markers with altered expression over the course of 52 weeks in the placebo group: CD16 and KIR2DS4 (Supplementary Figure S3B), which did not change in the daclizumab beta treated group. This suggests that the expression changes in 10 markers observed after 52 weeks of daclizumab beta treatment were due to the treatment rather than normal drift in marker expression over a year.

Within the daclizumab beta-treated group, most protein expression changes that occurred in total NK cells by 24 weeks were preserved at 52 weeks. CD56, NKp44, NKG2A, and CD2 predicted 24 weeks of treatment, while HLA-DR and FAS-L predicted baseline samples (Figure 1B). With the increased power from these paired comparisons, the changes in CD56, NKp44, NKG2A, CD2, and HLA-DR were significant after correction for multiple comparisons. When comparing baseline and 52 weeks, CD56, NKp44, NKG2A, and CD2 significantly predicted 52 weeks of treatment, while CD244, FAS-L, HLA-DR, NTB-A, and NKG2C significantly predicted baseline samples (Figure 1B).

CD56 was the most significant predictor of daclizumab beta treatment, and predicted both 24- and 52-week samples compared to baseline. Using UMAP visualization, we found an increase in frequency of CD56^bright^ NK cells by 24 weeks of daclizumab beta treatment, which continued to increase at 52 weeks (Figure 1C). This increase in CD56^bright^ NK cells was confirmed by gating in FlowJo (Figure 1D and Supplementary Figure S1B), which showed that the frequency of CD56^bright^ NK cells was increased in daclizumab beta-treated subjects compared to placebo-treated, and increased from baseline to 24 weeks and 52 weeks. There was no significant difference in frequency of CD56^bright^ NK cells in placebo-treated individuals over time, or in individuals with MS before treatment compared to healthy controls (Figure 1D).

Both the *CytoGLMM* analysis and UMAP visualizations revealed an increase in the CD56^bright^ population of NK cells upon daclizumab beta treatment. In order to test whether the CD56^bright^ population clustered distinctly from other NK cells, we used the CATALYST package to perform clustering of the NK cells. We determined that there were 5 metaclusters of NK cells in our data (Supplementary Figure S4A). These clusters varied in frequencies and marker expression (Supplementary Figure S4B). We performed differential abundance tests between the clusters to determine which clusters were more or less abundant at 52 weeks of daclizumab beta treatment compared to placebo (Supplementary Figure S4C). Two clusters showed differential abundance; cluster 5, representing 19.2% of NK cells, was less abundant upon 52 weeks of daclizumab beta treatment, and had high expression of CD16 and CD57. Cluster 2, representing 46.0% of total NK cells, had significantly higher frequency upon 52 weeks of daclizumab beta treatment, and had high expression of CD56 and CD2, and low expression of CD57 and HLA-DR. This analysis confirms that the CD56^bright^ population is the most significantly altered subset of NK cells upon daclizumab beta treatment.

### Daclizumab Beta Alters Expression of NK Receptors on the CD56^bright^ Population

**FIGURE 2:**
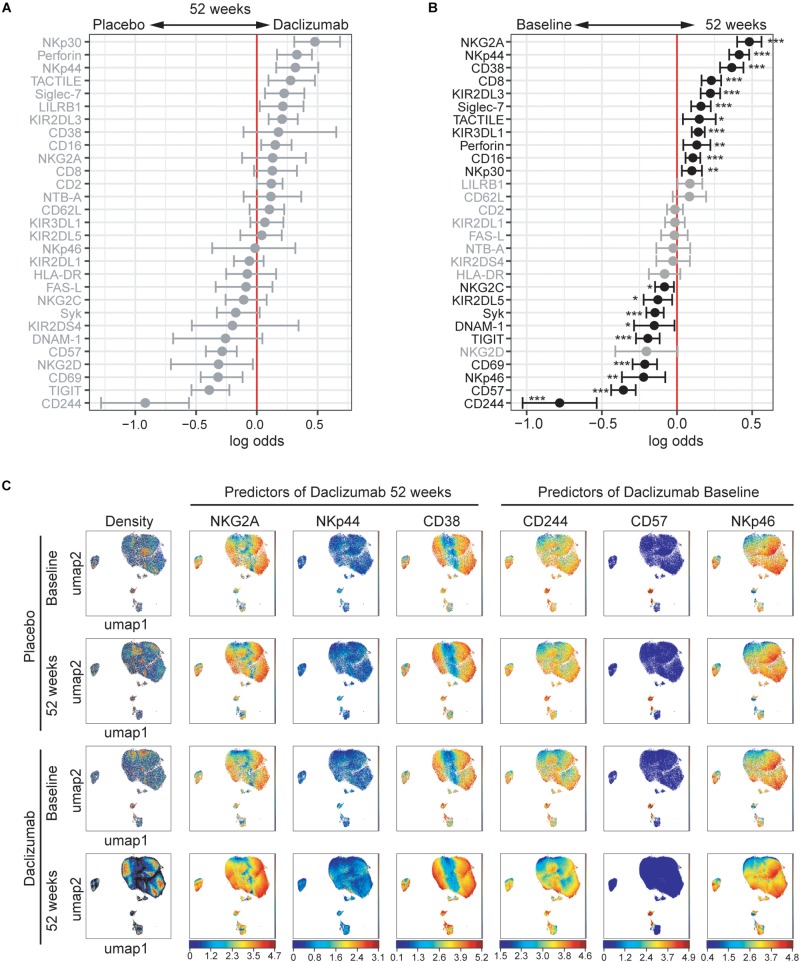
Daclizumab beta alters the CD56^bright^ population. **(A)** A generalized linear model with bootstrap resampling was used to identify NK markers on CD56^bright^ NK cells predictive of daclizumab beta- and placebo-treated individuals at 52 weeks of treatment. CD56^bright^ NK cells were used with no subsampling. Placebo: 24 weeks, *n* = 14; 52 weeks, *n* = 16. Daclizumab beta: 24 weeks, *n* = 25; 52 weeks, *n* = 27. Gray lines indicate markers with adjusted p-values > 0.05. Black lines indicate markers with adjusted *p*-values < 0.05. **(B)** A generalized linear mixed model with paired comparison was used for analyses of the same individual over time, comparing baseline and 52 weeks of daclizumab beta treatment. CD56^bright^ NK cells were used with no subsampling. Daclizumab beta baseline vs. 52 weeks, *n* = 22. Gray lines indicate markers with adjusted p-values > 0.05. Black lines indicate markers with adjusted *p*-values < 0.05. **(C)** UMAP visualizations of CD56^bright^ NK cells from the placebo at baseline (top row) and 52 weeks (second row), or the daclizumab beta-treated at baseline (third row) and 52 weeks (bottom row) groups. Leftmost panels are colored by cell density. NKG2A, NKp44, and CD38 were predictors of 52 weeks of daclizumab beta treatment. CD244, CD57, and NKp46 were predictors of baseline samples. Each plot is colored by marker expression, with color scale consistent between groups but specific for each marker. *Adjusted *p*-value < 0.05, **adjusted *p*-value < 0.01, ***adjusted *p*-value < 0.001. Adjusted *p*-values calculated using Benjamini-Hochberg method with FDR = 0.05.

As the CD56^bright^ and CD56^dim^ NK cell subsets are distinct, we next focused solely on the CD56^bright^ NK cells (Supplementary Figure S1B for gating strategy). The *CytoGLMM* package was used to identify which markers predicted daclizumab beta treatment compared to placebo within the CD56^bright^ population at 52 weeks. NKp30, Perforin, NKp44, TACTILE, Siglec-7, KIR2DL3, and CD16 predicted daclizumab beta treatment compared to placebo after 52 weeks of treatment in the unadjusted comparison, but were not significant following adjustment for multiple comparisons (Figure 2A).

In order to determine what changes in NK receptor expression occurred in response to daclizumab beta in treated individuals over time, the predictors of baseline or 52 weeks of treatment were determined using *CytoGLMM* (Figure 2B). Many NK receptors significantly predicted 52 weeks of daclizumab beta treatment: NKG2A, NKp44, CD38, CD8, KIR2DL3, Siglec-7, TACTILE, KIR3DL1, Perforin, NKp30, and CD16 (Figure 2B). CD244, CD57, NKp46, CD69, TIGIT, DNAM-1, Syk, KIR2DL5, and NKG2C significantly predicted baseline samples compared to 52 weeks.

Expression of each of the top three predictors of 52 weeks of daclizumab beta treatment and the top three predictors of placebo treatment was visualized using UMAP in both placebo and daclizumab beta treated groups at baseline and 52 weeks (Figure 2C). In the UMAP projections, the plots show an overall increase in density of CD56^bright^ NK cells at 52 weeks of daclizumab beta treatment (Figure 2C). NKG2A has higher expression in the CD56^bright^ population of the daclizumab beta treated group, and particularly higher expression in the areas of high cell density. Conversely, CD244 predicted placebo treatment, and had higher expression across the CD56^bright^ population of placebo-treated individuals compared to daclizumab beta-treated individuals. The UMAP plots reveal broad changes in expression of each of these markers across the CD56^bright^ population, as opposed to small subsets of CD56^bright^ cells with altered NK marker expression.

The mean signal intensity was calculated for each of the top six predictors of 52 weeks of daclizumab beta treatment and the top six predictors of baseline samples in each individual (Supplementary Figure S5). NKG2A and NKp44 expression increased in almost every individual by 24 weeks of daclizumab beta treatment, and tended to stay elevated at 52 weeks. CD38, CD8, KIR2DL3, and Siglec-7 expression increased in some but not all subjects, suggesting that the daclizumab beta-induced CD56^bright^ population is not equivalent in all subjects receiving daclizumab beta. CD244, CD57, NKp46, CD69, and TIGIT expression were significantly decreased by 24 weeks of daclizumab beta treatment in nearly all subjects, while NKG2D expression varied between individuals. These graphs highlight the fact that most of the changes in NK receptor expression observed with daclizumab beta treatment occur within the first 6 months of treatment, and are maintained throughout the course of treatment.

### Daclizumab Beta Treatment Does Not Alter a Specific Subset of CD56^bright^ NK Cells

**FIGURE 3:**
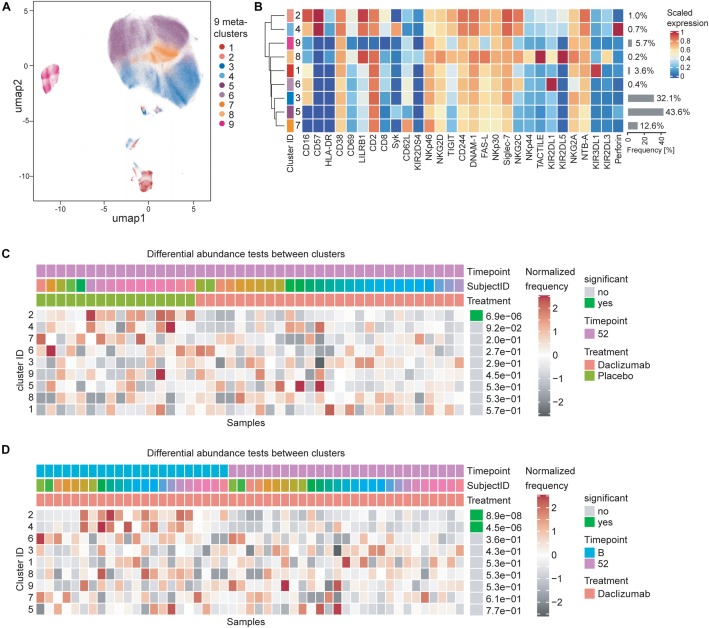
Daclizumab beta treatment does not alter a specific subset of CD56^bright^ NK cells. **(A)** The CATALYST package was used to perform clustering on all CD56^bright^ NK cells from both placebo and daclizumab beta treated groups at all time points. Using the delta area plot provided by the clustering analysis, 9 metaclusters were retained. The metaclusters are displayed in the UMAP space, and colored by metacluster. **(B)** The heatmap shows the cluster IDs (leftmost column) hierarchically ordered by similarity (dendrogram calculated using Euclidean distance as a metric and average as a linkage). Markers are labeled at the bottom of the heatmap. The marker expressions are scaled to values between 0 and 1. Along the right side of the heatmap, the frequency of each cluster among the total CD56^bright^ NK population is shown in gray bars, with the frequency printed next to it. **(C)** Differential abundance test between clusters at 52 weeks of treatment comparing placebo and daclizumab beta treatment. Across the top, Timepoint, Subject ID, and Treatment are shown for each sample (columns). The heatmap represents the proportion of each metacluster in each sample, with gray showing under-representation and red showing over-representation. The proportions are first scaled with arcsine-square-root transformation and then z-score normalized in each cluster. The cluster ID is labeled along the left side of the heatmap, ordered by significance, which is shown along the right side of the heatmap. The differential abundance test reports adjusted *p*-values (FDR). **(D)** Differential abundance test between clusters within the daclizumab beta treated group, comparing baseline and 52 weeks of treatment. Across the top, Timepoint, Subject ID, and Treatment are shown for each sample (columns). The cluster ID is labeled along the left side of the heatmap, ordered by significance, which is shown along the right side of the heatmap. The differential abundance test reports adjusted *p*-values (FDR).

In order to test whether the NK marker expression changes observed upon daclizumab beta treatment were due to changes in a particular subset of CD56^bright^ NK cells, we performed clustering analysis using the CATALYST package. We determined that there were 9 metaclusters of CD56^bright^ NK cells (Figure 3A) that varied in metacluster frequency and marker expression (Figure 3B). We performed a differential abundance test between clusters in the daclizumab beta and placebo groups at the 52-week time point (Figure 3C). This test revealed one cluster that was significantly less abundant in daclizumab beta-treated individuals; cluster 2, only representing 1.0% of CD56^bright^ NK cells, had high expression of CD16, CD57, and LILRB1, and low expression of Syk and Perforin. We performed a differential abundance test between clusters in the daclizumab beta-treated group comparing baseline and 52 weeks of treatment, and found two clusters with significantly altered frequency; both cluster 2, again representing only 1.0% of NK cells, and cluster 4, representing only 0.7% of NK cells, showed lower abundance at 52 weeks than at baseline (Figure 3D). Cluster 4 had high expression of CD57 and Perforin. While these analyses do reveal significant differences in two subsets of CD56^bright^ NK cells upon daclizumab beta treatment, the clusters that have differential abundance represent such a small portion of NK cells that we conclude that the alterations we observed in NK receptor expression occur broadly across the CD56^bright^ population, rather than as a result of a significant shift from one CD56^bright^ subset to another.

### Daclizumab Beta Also Alters NK Receptor Expression in the CD56^dim^ Population

**FIGURE 4:**
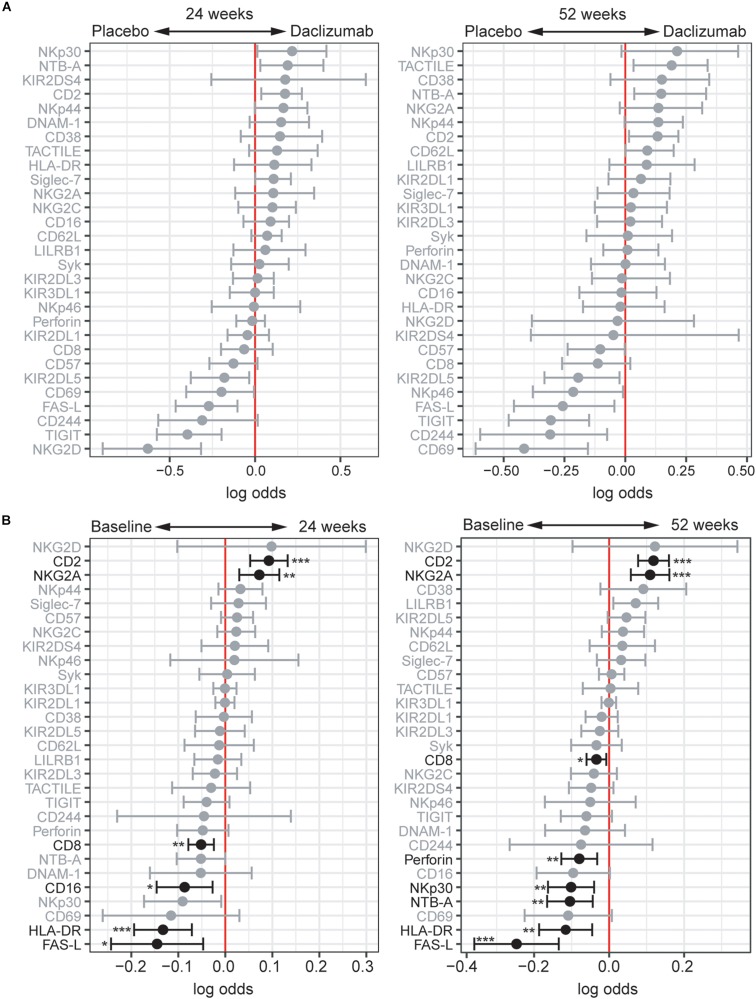
Daclizumab beta changes NK receptor expression on CD56^dim^ cells distinctly from CD56^bright^ cells. **(A)** A generalized linear model with bootstrap resampling was used to identify NK markers on CD56^dim^ NK cells predictive of daclizumab beta- and placebo-treated individuals at 24 (left) and 52 (right) weeks of treatment. CD56^dim^ NK cells were used with subsampling to 1000 cells per individual. Placebo: 24 weeks, *n* = 14; 52 weeks, *n* = 16. Daclizumab beta: 24 weeks, *n* = 25; 52 weeks, *n* = 27. Gray lines indicate markers with adjusted *p*-values > 0.05. Black lines indicate markers with adjusted *p*-values < 0.05. **(B)** A generalized linear mixed model with paired comparison was used for analyses of the same individual over time, comparing baseline and 24 weeks of daclizumab beta treatment (left), and baseline and 52 weeks of daclizumab beta treatment (right). CD56^dim^ NK cells were used with no subsampling. Daclizumab beta baseline vs. 24 weeks, *n* = 21. Daclizumab beta baseline vs. 52 weeks, *n* = 22. *Adjusted *p*-value < 0.05, **adjusted *p*-value < 0.01, ***adjusted *p*-value < 0.001. Adjusted *p*-values calculated using Benjamini-Hochberg method with FDR = 0.05.

While the focus of this study was to determine NK receptor expression in the daclizumab beta-induced CD56^bright^ population, it was interesting to find that there were some distinct changes in NK receptor expression observed in the CD56^dim^ population as well. Using *CytoGLMM*, we identified several weak (low log-odds) predictors of daclizumab beta treatment compared to placebo in the CD56^dim^ population in the unadjusted analysis, including NTB-A and CD2 at 24 weeks, and TACTILE, NTB-A, and CD2 at 52 weeks; these findings were not significant after correcting for multiple comparisons (Figure 4A). There were stronger (higher log-odds) predictors of placebo treatment at both 24 and 52 weeks, including NKG2D, TIGIT, FAS-L, KIR2DL5, CD69, CD244, and NKp46 in the unadjusted analysis (Figure 4A). This suggests that daclizumab beta more strongly decreased expression of several NK receptors in the CD56^dim^ population rather than increased.

When comparing baseline and 24 or 52 weeks of treatment in daclizumab beta-treated individuals, we identified two significant predictors of treatment: CD2 and NKG2A (Figure 4B). Conversely, FAS-L, HLA-DR, CD16, CD8, NTB-A, NKp30, and Perforin predicted baseline samples compared to 24 or 52 weeks. Interestingly, several of the markers that predicted baseline samples in the CD56^dim^ population actually predicted daclizumab beta treatment in the CD56^bright^ population, suggesting that distinct responses to daclizumab beta treatment occur in the CD56^bright^ and CD56^dim^ NK cells.

## Discussion

While daclizumab beta has been voluntarily withdrawn from the market, understanding the effects of daclizumab beta may provide insight into the role of NK cells in the pathogenesis of RMS and in the potential of other NK cell-based therapeutic strategies for RMS. Here, we used mass cytometry to profile the NK cells expanded in the setting of daclizumab beta treatment of RMS. As observed previously (10), daclizumab beta led to a dramatic shift in the NK cell repertoire with an increase in the frequency of CD56^bright^ NK cells and enhanced expression of NKG2A and CD2. Here, we extended the characterization of the phenotype, demonstrating that the expanded CD56^bright^ NK cells had a unique phenotype that was present by 24 weeks of treatment and remained at 52 weeks of treatment.

Daclizumab beta treatment was associated with enhanced expression of the activation markers CD38 and Perforin, the activating receptors NKp44, TACTILE (CD96), and CD16, and the inhibitory receptors NKG2A, KIR2DL3, and Siglec-7 within the CD56^bright^ population. The enhanced expression of a number of markers of cellular activation and receptors that mediate NK cell activation suggests that the CD56^bright^ NK cells that emerge upon daclizumab beta treatment are primed for responsiveness. At the same time, it is difficult to fully predict the effects of these alterations in receptor expression profiles. While CD56^bright^ NK cells normally express NKG2A, the inhibitory receptor that binds HLA-E, this expression is even higher following daclizumab beta treatment (Figure 2). While enhanced expression of this inhibitory receptor could diminish NK cell responsiveness, it is also possible that these NK cells are “educated” through NKG2A, leading to their enhanced ability to detect “altered self” (37, 38). Along similar lines, the increased expression of KIR2DL3 on the CD56^bright^ NK cells in daclizumab beta-treated subjects indicates that at least a subset of these CD56^bright^ NK cells have a more mature phenotype, and could be educated through this KIR to enhance their ability to respond to missing self. Thus, daclizumab beta treatment drives several phenotypic changes in NK cells that could contribute to enhanced NK cell responsiveness.

Daclizumab beta treatment had less effect on the mature CD56^dim^ subset that is generally associated with higher cytolytic activity, yet phenotypic changes still occurred. Similar to CD56^bright^ NK cells, expression of NKG2A and CD2 on CD56^dim^ cells was predictive of daclizumab beta treatment. Interestingly, several markers associated with cellular cytotoxicity, including FAS-L and Perforin, predicted baseline samples among CD56^dim^ NK cells, suggesting that the CD56^dim^ NK cells from daclizumab beta treated subjects may be less efficient at killing. This could partially explain the killing of autoreactive T cells by CD56^bright^ NK in the setting of daclizumab beta treatment (10).

In general, CD56^bright^ NK cells are thought of as the immature subset of NK cells that do not express KIRs, have limited cytolytic activity, and secrete cytokines. Their expansion following daclizumab beta treatment could therefore improve outcomes in RMS through either immunomodulation by cytokine secretion or by killing autoreactive T cells. Surprisingly, prior work demonstrated that the daclizumab beta-expanded CD56^bright^ NK cells can kill activated autologous CD4^+^ T cells in a granzyme K dependent manner *in vitro* (10, 20), which may well explain the therapeutic effect of daclizumab beta. The fact that there was a strong correlation between CD56^bright^ NK cell expansion and T cell contraction following daclizumab beta treatment supports the idea that the CD56^bright^ NK cells could be limiting disease by eliminating T cells *in vivo* (10). One potential explanation for this surprising cytotoxicity mediated by CD56^bright^ NK cells is that they are not “conventional” CD56^bright^ NK cells, but instead fully mature CD56^dim^ NK cells that upregulated CD56 expression to the point where they were re-classified as CD56^bright^ NK cells. In fact, prior work demonstrates that IL-2 increases CD56 expression on NK cells (7). However, as visualized by UMAP, the expanded NK cells in daclizumab beta-treated individuals cluster in the same region as CD56^bright^ NK cells from placebo-treated individuals, not with CD56^dim^ NK cells (Supplementary Figure S4). Instead, the CD56^bright^ NK cells appear to have acquired key characteristics that could enhance their cytolytic activity with daclizumab beta treatment, including enhanced expression of Perforin and CD16. Further, the daclizumab beta-induced CD56^bright^ NK cells express other markers of maturity, including KIR2DL3, Siglec-7, CD38, and CD8. Together, these data are consistent with the idea that the CD56^bright^ NK cells induced by daclizumab beta treatment are activated and have acquired sufficient maturity to be cytolytic.

Prior studies have indicated that NK cells may play a role in MS pathogenesis in the absence of drug treatment. Two groups have reported that CD56^bright^ NK cells are present in the CNS in MS lesions (39, 40), suggesting they may play a role in eliminating activated CD4^+^ T cells in brain lesions. Martinez-Rodriguez et al. reported that CMV infection, which drives an expansion of mature, “adaptive” NKG2C-expressing NK cells, is associated with a lower risk of disease progression in MS (41). The CD56^bright^ NK cells observed after daclizumab beta do not resemble the mature NKG2C-expressing NK cells seen after CMV infection, but nonetheless could contribute to protection within the CNS.

Gross et al. report that CD56^bright^ NK cells are present in CNS lesions in MS, but that NK cells from MS patients are deficient in killing autologous, activated CD4 + T cells (40). This defect is attributed to poor expression of DNAM-1 on NK cells and its ligand, CD155 on CD4^+^ T cells in the setting of MS (40), and is consistent with studies revealing that DNAM-1 polymorphisms may play a role in susceptibility to MS (42). Independently, CD56^bright^ NK cells have been reported to mediate killing through NKG2D, TRAIL, and LFA-1 expression (43). In our study of peripheral blood NK cells, DNAM-1 expression was predictive of baseline samples, indicating that DNAM-1 expression is not increased by daclizumab beta treatment. However, it is important to note that our study characterized peripheral blood NK cells, and the prior studies showing DNAM-1-mediated killing were all focused on NK cells in the CNS.

Several studies suggest that NK cells may be deficient in the setting of MS (44–50), providing hope that enhancing their frequency and/or function could improve outcomes. However, some studies have not demonstrated a defect in NK cell numbers in MS subjects (40, 51). Another study suggests that ‘regulatory’ NK cells may be more important in disease pathogenesis (52, 54). A limitation of our study was that our control group of healthy controls was collected independently of the SELECT and DECIDE trials. For comparison of CD56^bright^ frequencies, we used local healthy blood bank control subjects, but the potential for batch effects based on collection of blood samples at different times and with different methods (e.g., CPT tubes vs. heparin tubes), precludes our ability to compare NK cells between RMS subjects and healthy controls without concern for batch effects.

There are several limitations to our study. The first is that the sample size is quite modest, leading to reduced power to find differences in this high-dimensional analysis, particularly in the cross-sectional comparison between daclizumab beta-treated and placebo-treated individuals. Second, as discussed above, we did not have a healthy control group to which we could directly compare NK cells in RMS patients without concern for batch effects. Third, we did not perform functional assessments, so cannot confirm that the enhanced expression of killing and activation markers on CD56^bright^ NK cells in fact drives better killing of autologous, activated CD4^+^ T cells. It is important to note that enhanced killing of CD4^+^ T cells was demonstrated previously in daclizumab beta treated subjects (10, 20).

Overall, these data demonstrate that significant changes occur in NK cells in response to the increased IL-2 availability induced by daclizumab beta treatment. These data extend prior findings indicating that daclizumab beta treatment increases the frequency of the CD56^bright^ population, which is not due to an increase of a specific subset of CD56^bright^ NK cells. NK receptor expression is broadly altered across CD56^bright^ NK cells, highlighting the unique phenotypic features of these expanded NK cells. The high expression of activation markers, activating receptors, and perforin could enhance the ability of these CD56^bright^ NK cells to control RMS through cytolytic activity or other immunoregulatory functions. The deep profiling of NK cells performed here, including in the placebo group, can also serve as a reference for future studies of NK cell phenotype in the setting of RMS. While daclizumab beta was voluntarily withdrawn from the market due to serious adverse events, it is notable that several other medications used to treat RMS, including natalizumab, fingolimod, glatiramer acetate, or beta interferon, are also associated with expanded CD56^bright^ NK cells in the setting of clinical response (53). Thus, in order to improve treatment for RMS, it will be critical in future studies to determine whether specific features of NK cells are associated with clinical response or serious adverse events.

## Data Availability Statement

The datasets generated for this study are available on request to the corresponding author. Our data is publicly available in flow repository (http://flowrepository.org/id/FR-FCM-Z2D6).

## Ethics Statement

This study used samples from two clinical trials: NCT00390221 and NCT01064401. We received de-identified patient data and blood samples. The patients/participants provided their written informed consent to participate in this study.

## Author Contributions

CB and JF conceptualized this study. TR and CB designed the experiments. TR and EV conducted the experiments. TR, LS, and NZ analyzed the data with statistical analysis input from CS, A-MF, and SH. LS, TR, and CB wrote the manuscript. All authors contributed to the revision of the manuscript.

## Conflict of Interest

CB received funding from Biogen to perform this study. JF was employed by Biogen Idec when the study was initiated and is currently employed by Sangamo Therapeutics. Biogen played no role in the analysis or interpretation of data. The remaining authors declare that the research was conducted in the absence of any commercial or financial relationships that could be construed as a potential conflict of interest.

## Footnotes

1 https://www.r-project.org/
